# Digital health applications and the fast-track pathway to public health coverage in Germany: challenges and opportunities based on first results

**DOI:** 10.1186/s12913-022-08500-6

**Published:** 2022-09-21

**Authors:** Hendrikje Lantzsch, Helene Eckhardt, Alessandro Campione, Reinhard Busse, Cornelia Henschke

**Affiliations:** 1 Department of Health Care Management, Technische Universität Berlin, Straße des 17. Juni 135, 10623 Berlin, Germany; 2 Technische Universität Berlin, Berlin Centre of Health Economics Research, Berlin, Germany

**Keywords:** Digital health applications, Digital health technology, DiHA, DiGA, ehealth, Evidence evaluation, Evaluation concept, Classification

## Abstract

**Objectives:**

Evidence-based decision-making is the sine qua non for safe and effective patient care and the long-term functioning of health systems. Since 2020 Digital Health Applications (DiHA, German DiGA) in Germany have been undergoing a systematic pathway to be reimbursed by statutory health insurance (SHI) which is attracting attention in other European countries. We therefore investigate coverage decisions on DiHA and the underlying evidence on health care effects, which legally include both medical outcomes and patient-centred structural and procedural outcomes.

**Methods:**

Based on publicly available data of the Institute for Medicines and Medical Devices searched between 08/2021 and 02/2022, all DiHA listed in the corresponding registry and thus reimbursable by the SHI were systematically investigated and presented descriptively on the basis of predefined criteria, such as clinical condition, and costs. The clinical trials on DiHA permanently included in the registry were reviewed with regard to their study design, endpoints investigated, the survey instruments used, and whether an intention-to-treat analysis was performed. Risk of bias was assessed using the ROB II tool.

**Results:**

By February 2022, 30 DiHA had been included in the DiHA registry, one third of them permanently and two thirds conditionally. Most DiHA were therapeutic applications for mental illness based on cognitive behavioural therapy. For all permanently included DiHA, randomised controlled trials were conducted to demonstrate the impact on health care effects. While medical outcomes were investigated for all of these DiHA, patient-centred structural and procedural outcomes were rarely investigated. The majority of clinical trials showed a high risk of bias, mainly due to insufficient reporting quality. Overall, the prices for DiHA covered by SHI are on average around € 150 per month (min. € 40; max. € 248).

**Conclusions:**

Evidence-based decision-making on coverage of DiHA leaves room for improvements both in terms of reporting-quality and the use of patient-centred structural and procedural outcomes in addition to medical outcomes. With appropriate evidence, DiHA can offer an opportunity as an adjunct to existing therapy while currently the high risk of bias of the trials raises doubts about the justification of its high costs.

**Supplementary Information:**

The online version contains supplementary material available at 10.1186/s12913-022-08500-6.

## Background

Given limited health professional resources and limited (spatial) access in rural areas, DiHA is seen as having the potential to improve health care delivery [1–3]. DiHA includes cooperative or interactive applications of modern information and communication technologies aiming to improve health care provision and population health. However, especially in the light of evidence-based health care, assessing DiHA is crucial to ensure safe, efficacious and effective health care. In Europe, the requirements under the Medical Device Regulation (MDR) to obtain market authorization (Conformité Européenne, CE) do not require proof of efficacy and effectiveness for DiHA that fall into the lower risk classes I and II. Uncertainty regarding benefits of DiHA is thus high, especially in the context of coverage decisions. This may also lead to a partially reserved acceptance among physicians, for instance in Germany [4].

Although Germany has been less advanced in terms of digitisation in health care compared to other countries so far [5], and digitisation is also insufficient in the hospital sector [6], it was the first country to launch a DiHA “fast-track” pathway for coverage decisions of digital health applications in ambulatory care. This has led to changes advancing development and evaluation of DiHA [7]. Germany has been a pioneer for many countries but the use of DiHA is also increasingly supported by the governments of other European countries [8]. France, for instance, will follow suit to foster innovation and provide access to DiHA for patients [9]. Many countries are currently developing national digital health strategies to include DiHA into the benefit basket of public health systems [10]. Several approaches for categorisation and decision making whether to include DiHA in public benefit baskets exist in European countries. Examples include an approach for categorisation, evaluation, and pricing/reimbursement developed to support of the German “Digital Health Care” (DVG) legislative process [11], the “Evidence standards framework for digital health technologies” concept developed by the National Institute for Health and Care Excellence (NHS) of England [12], a platform and a validation pyramid for CE-marked mobile applications in Belgium [13], and the French guide on clinical evaluation of a medical device in the context of eligibility decisions for reimbursement [14] complemented by a French Health Authority (HAS) system for classification of digital solutions according to their intended use, their ability to provide an individual response and their autonomy [15]. However, the evidence base for coverage decisions is a frequently and highly discussed topic. Increasingly, models are being developed to evaluate DiHA [16–18] also due to methodological gaps and difficulties in assessing the value of DiHA, including the question for an adequate control group to be included in clinical trials and the level of evidence to be used [19].

Although a process of assessing DiHA with regard to coverage decisions was set up in Germany in 2019, there is no study analysing its findings. Therefore, this study aims to investigate the first results of the fast-track pathway while especially focusing on the evidence evaluation of DiHA. Additionally, risk of bias of clinical trials used in the decision-making process of the German fast-track pathway was assessed.

### Procedure and reimbursement of DiHA in Germany

In Germany, the Digital Health Care Act (Digitale-Versorgung-Gesetz, DVG) was passed in November 2019 [20]. It describes the formalisation of the DiHA, which can be prescribed by physicians and psychotherapists or reimbursed directly by sickness funds upon request of insured persons provided the corresponding diagnoses was made by the physician. DiHA may include standard software, software as a service, mobile as well as browser-based applications. Insureds are entitled to certain DiHA, which must meet certain requirements in order to be covered by the German statutory health insurance (SHI).

**Fig. 1:**
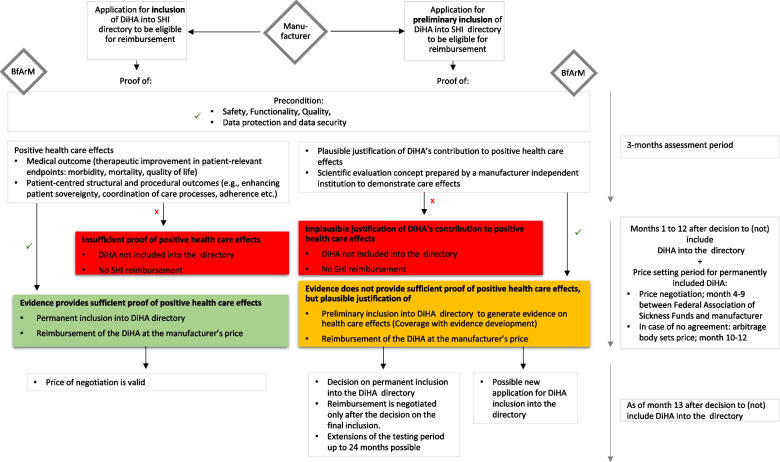
Overview of process for digital health applications under the DVG (own illustration)

The subsequent Ordinance on Digital Health Applications (Digitale Gesundheitsanwendungen-Verordnung, DiGAV) describes the procedure [21]. DiHA-manufacturers must apply to the German Institute for Medicines and Medical Devices (Bundesinstitut für Arzneimittel und Medizinprodukte, BfArM) to be considered for coverage through SHI. Their application must meet requirements for safety, quality, functionality, privacy, and data security. Furthermore, evidence of positive health care effects must be shown. The latter include medical outcomes and patientcentred structural and procedural outcomes to be demonstrated by corresponding endpoints in comparative clinical trials. The manufacturer must decide between two possibilities of the fast-track pathway: an application to be considered (1) permanently or (2) conditionally into SHI directory to be eligible for reimbursement. (1) If evidence of positive health care effects is available at the time of application, the DiHA can be included in the official DiHA directory of SHI. (2) For DiHA following the application process to be conditionally included into the official directory for a limited time period (usually twelve months, max. 24 months), manufacturers have to submit plausible justification of DiHA’s contribution to positive health care effects and a scientific evaluation concept prepared by a manufacturer-independent institution to demonstrate health care effects. Following the concept of coverage with evidence development, manufacturers must generate respective evidence of positive health care effects, while the DiHA is conditionally reimbursed (Fig. 1). After inclusion in the directory, manufacturers are basically free to set their own prices for the first year. From the 13th month after inclusion into the DiHA directory, the price negotiated between the manufacturer and Federal Association of Sickness Funds applies.

## Methods

**Fig. 2:**
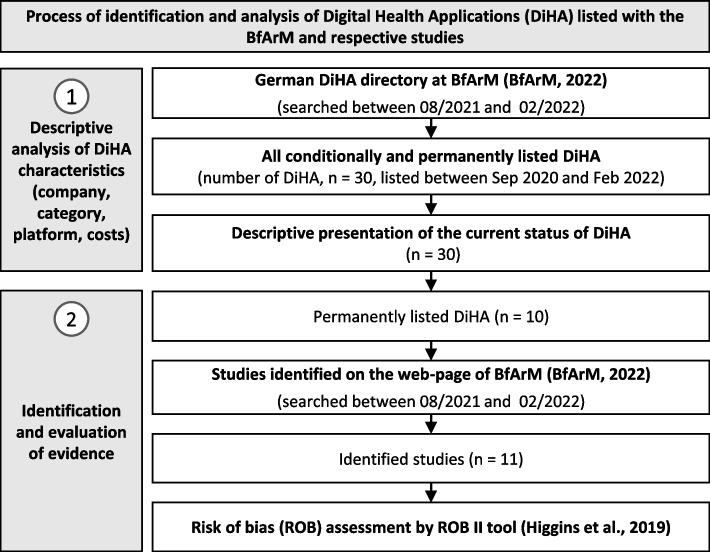
Methodological approach (own illustration)

Figure 2 gives an overview on the methods of the study. We identified between 08/2021 and 02/2022 all DiHA listed in the DiHA directory and respective decision documents of BfArM [22] used during the fast-track evaluation between 09/2020, when the first decisions were made, and 02/2022.

First, all DiHA permanently and conditionally included in the German DiHA directory were investigated descriptively according to predefined criteria: name of DiHA, registry inclusion, company, platform and price details. Data were extracted from the publicly available data sources of the BfArM [22]. Prices represent costs covered by SHI and were analysed descriptively and presented graphically in a boxplot.

Second, clinical trials on the permanently included DiHA identified in the respective documents of BfArM (summary section: information on positive health care effects) for each DiHA [22] were examined with regard to the study design, the endpoints investigated and the survey instruments used, and whether an intention-to-treat analysis was performed. In addition, data was extracted on diagnostic category and health problem, type and level of evidence of the identified trial, the number of trial participants randomised, type of interventions in experimental and control arm, planned time points of the longest follow-up, and evaluated outcomes and survey instruments used for the measurement of the outcome. The level of evidence (LoE) of identified clinical trials was determined based on classification according to chapter 2, Sect. 3, § 11, Nr. 2 of the rules of procedure of the Federal Joint Committee [23]. Data availability on evidence for DiHA conditionally included was analysed descriptively and classified according to the information in the DiHA directory [22]. The data extraction was performed by two persons (HL and AC).

Third, the risk of bias in the identified randomised controlled trials (RCT) was assessed independently by two researchers (HL, HE) using the revised Cochrane risk of bias tool for randomised trials (RoB II) [24]. Disagreements during the assessment were discussed until agreement was reached. The ROB II tool covers five domains that can introduce bias. The domains consider aspects of trial design, conduct, and reporting. The domains “randomisation process” and the “effect of assignment to an intervention” were assessed at trial level, while the domains “missing outcome data”, “measurement of the outcome” and the “selection of reported results” were assessed at outcome level (morbidity) of each trial. Each domain is subdivided into several aspects and each aspect is assessed against a series of questions that help to identify the risk of bias. The assessment of each aspect is finally combined into a judgement for one domain and for the entire trial at the outcome level [25]. To assess ROB II, all available publications on a trial, trial protocols and trial registry entries, if available, were used.

## Results

### Overview of DiHA included in the German DiHA directory

**Fig. 3:**
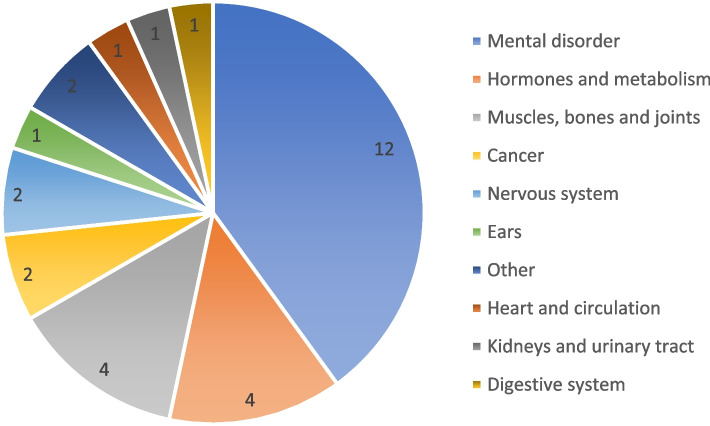
Clinical conditions of reimbursable DiHA, *n* = 30 (own illustration; data base: BfArM, 2022b)

As of February 2022, 30 digital health applications were listed in the DiHA directory of BfArM. The majority of the DiHA (*n* = 20/30) had been conditionally included while ten DiHA were listed permanently [22]. Additional file 1 gives an overview on DiHA listed in the directory. Two-thirds of DiHA were admitted in 2021 (*n* = 18/30), one-third in 2020 (*n* = 10/30) and two DiHA in early 2022. The manufacturers are most often start-up companies, small to medium-sized companies. Employee numbers range up to 140. Almost half (*n* = 14/30) of the DiHA are web-only applications, 13 of 30 DiHA are Apple iOS and Google Android applications only and three of 30 DiHA are accessible via of these three platforms. Most DiHA (*n* = 19/30) applied the concept of cognitive behavioural therapy in the therapeutic field of mental diseases and disorders [22]. However, the conditions targeted by the DiHA are diverse, ranging from cognitive behavioural therapy for tinnitus or alcohol dependence, to the application of individual elements of behavioural therapy for diabetes self-management (Fig. 3).

For a few DiHA (*n* = 4/30), there may be additional costs to be paid by the user if they wish to use certain additional services that are not covered by the prescription. Two thirds of the DiHA (*n* = 22/30) were only available in German language; only some in one additional language (*n* = 4/30) and several languages (*n* = 4/30) [22].

**Fig. 4:**
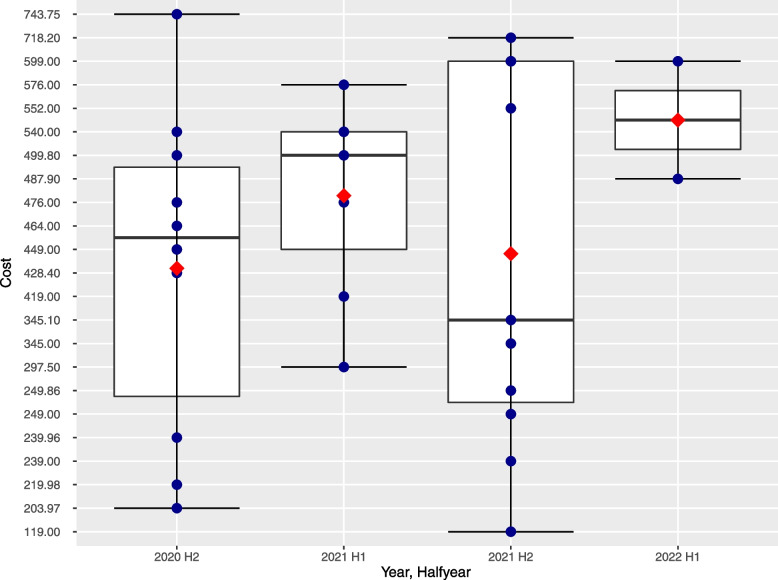
Quarterly cost of DiHA at the time of inclusion in the DiHA registry (own illustration)

The costs of DiHA covered by SHI are listed in the directory. Those include costs of start-up package and costs for the use of the first 90 days.In some cases, there are cost for an additional 90 days of use (*n* = 4/30). Overall, the average price for the first 90 days is € 443.80 per quarter with a price spectrum ranging from € 119.00 to € 743.75. The most expensive DiHA thus cost 6.3 times as much as the cheapest. The average cost of a DiHA permanently listed is € 469.82 per first quarter (min = € 203.97, max = € 743.75). The average cost of the DiHA listed conditionally is € 430.80 per first quarter (min = € 119.00, max = € 718.20). No extra payments by patients are required for any DiHA listed in the DiHA directory. Figure 4 shows the cost of all DiHA in time trend by half-year. The prices are widely distributed within each half-year and, on average, increase slightly over time.

### Data availability on evidence of health care effects for DiHA conditionally included in the registry

With the requirement to provide evidence of positive health care effects, 20 DiHA were provisionally listed (Additional file 1). For the DiHA CANKADO PRO-React Onco, an RCT is ongoing (NCT03220178). For the DiHA Novego, three RCTs were already published [26–28] and another RCT is currently ongoing. For the other DiHA, the current evidence is limited to efficacy data, systematic data evaluations, preliminary clinical trials, and “data from a sample” [22]. For all DiHA, an assessment of the medical outcome is planned. For five DiHA (CANKADO PRO-React Onco, Cara Care für Reizdarm, Kranus Edera, Mindable, Rehappy), an additional demonstration of patient-centred structural and procedural outcomes such as health literacy, patient sovereignty, adherence, and patient satisfaction is planned [22]. The manufacturers of the DiHA provisionally included state that they want to conduct an RCT (*n* = 19/20), and a multi-centre, prospective, two-arm study (Cankado) (*n* = 1/20).

### Data availability on evidence of health care effects for DiHA permanently included in the registry

Table 1 gives an overview on permanently listed DiHA (*n* = 10/30), and clinical trials used in the fast-track pathway. RCTs were conducted for all DiHA with two clinical trials still in the process of publication (Kalmeda and Vivira). The number of trial participants randomised was on average 281 (max = 1,013; min = 56). The intervention and control groups were always similar in number.

**Table 1:** Overview of the evidence of the permanently included DiHA; 09/2020 – 02/2022 (based on information of DiHA directory, clinical trials, and study registers)

**#**	**DiHA**^**a**^	**Diagnostic category / health problem**	**Author (year)**	**LoE**^**b**^ **(trial design)**	**Number of trial participants randomised** **(IG, CG)**	**Intervention in the experimental arm**	**Intervention in the control arm**	**Follow-up (planned, max, in months)**	**Reported outcomes**	
									**Medical outcomes**	**Patient-centred structural and procedural outcomes**
**1**	**deprexis**	Mental disorder / depression	Klein et al. (2016) + Klein et al. (2013) [29, 30]	Ib (RCT)	N = 1.013 (IG = 509, CG = 504)	Guided^c^ Web-based intervention (Deprexis)	Care as usual	6	Primary: depressive symptom severity measured by PHQ-9; Secondary: severity of depression, psychiatric diagnoses, acute suicidality measured by HDRS-24, QIDS-C16, PHQ-9; quality of life measured by SF-12	
		Mental disorder / depression	Meyer et al. (2015) [31]	Ib (RCT)	N = 163 (IG = 78, CG = 85)	Unguided^d^ Web-based intervention (Deprexis)	Waitlist	6	Primary: depressive symptom severity measured by PHQ-9; Secondary: anxiety symptoms, somatic complaints measured by GAD-7, PHQ-15; quality of life measured by SF-12	
		Mental disorder / depression	Berger et al. (2011) [32]	Ib (RCT)	N= 76 (IG1 = 25; IG2=25, CG = 26)	Unguided Web-based intervention (Deprexis); guided Web-based intervention (Deprexis)	Waitlist	6	Primary: depressive symptom severity measured by BDI-II; Secondary: psychological symptoms measured by 53-item BSI; interpersonal problems measured by IIP; quality of life measured by WHOQOL-BREF	
		Mental disorder / depression	Moritz et al., (2012) [26]	Ib (RCT)	N = 210 (IG = 105, CG = 105)	Unguided web-based intervention (Deprexis)	Waitlist	NA	Primary: depressive symptom severity measured by BDI; Secondary: acute suicidality measured by DAS, RSE, SBQ-R; quality of life measured by WHOQOL-BREF	
**2**	**elevida**	Nervous system / multiple sclerosis	Pöttgen et al. (2018) [33]	Ib (RCT)	N = 275 (IG = 139, CG = 136)	Unguided web-based intervention (elevida)	Waitlist	6	Primary: severity of physical and mental fatigue measured by Chalder Fatigue Scale; Secondary: MS-specific motor and cognitive fatigue, anxiety and depression, quality of life, self-reported cognitive difficulties, activities of daily living measured by FSMC, HADS-A and HADS-D, HAQUAMS, MSNQ	
**3**	**HelloBetter Diabetes und Depression**	Hormones and metabolism / diabetes mellitus, type 1 and 2	Nobis et al (2015) + Nobis et al. (2013) [34, 35]	Ib (RCT)	N = 260 (IG = 130, CG = 130)	Guided web- and mobile-based self-help intervention (GET.ON Mood Enhancer Diabetes)	Online unguided psychoeducation Program for depression	1	Primary: depressive symptom severity measured by CES-D; Secondary: behavioural activation, problem solving, worry, diabetes-related emotional stress, quality of life measured by HADS, PAID, AADQ, DSMQ, CSQ-8	
**4**	**HelloBetter Stress und Burnout**	Other / difficulties in coping with life	Heber et al. (2016) [36]	Ib (RCT)	N = 264 (IG = 132, CG = 132)	Guided web- and mobile-based training (GET.ON Stress)	Waitlist	12	Primary: stress exposure measured by PSS-10; Secondary: depressive symptoms, anxiety symptoms, emotional exhaustion, sleep (severity of insomniac symptoms), work engagement, self-assessment of emotional competencies, psychological distancing from work, worries measured by CES-D, ISI, HADS-A, PSWQ-PW, SF-12, UWES, REQ-PD, TiC-P-G, ERSQ-27, ERSQ-ES-GD, CSQ-8	
**5**	**HelloBetter Vaginismus Plus**	Mental disorder / non-organic vaginismus	Zarski et al. (2021) + Zarski et al. (2018) [37, 38]	Ib (RCT)	N = 200 (IG = 100, CG = 100)	Guided web-based intervention (eHealth platform)	Waitlist	6	Primary: improvement of vaginal penetration during sexual intercourse measured by PEQ; Secondary: non-coital vaginal penetration ability, sexual functioning, sexuality-related anxiety, penetration-related cognitions measured by PEQ, FSFI, FSQ, VPCQ	
**6**	**Kalmeda**	Ears / tinnitus	In publication process^e^	Ib (RCT)	N = 187	Mobile-app “Kalmeda”	Waitlist	3	Primary: tinnitus exposure measured by mini-TQ-12; Secondary: tinnitus burden, depression tendency, stress experience and self-efficacy measured by PHQ-9, PSQ20, SWOP-K9	
**7**	**somnio**	Mental disorder / insomnia	Lorenz et al. (2019) [39]	Ib (RCT)	N = 56 (IG = 29, CG = 27)	Unguided web-based therapy	Waitlist	12	Primary: insomnia severity measured by ISI, APSQ, SRBQ; Secondary: depression symptomatology, anxiety symptomatology, somatisation measured by BDI-II, BSI-Somatization	
**8**	**velibra**	Mental disorder / panic and anxiety disorders	Berger et al. (2017) [40]	Ib (RCT)	N = 139 (IG = 70, CG = 69)	Unguided web-based-based cognitive–behavioural treatment (velibra)	Waitlist	6	Primary: disorder unspecific measures of anxiety, depressive symptoms, tension/stress measured by DASS-21, BAI, BDI-II; general psychopathology measured by BSI; quality of life measured by SF-12; Secondary: disorder-specific measures of anxiety measured by SPS, SIAS, ACQ, BSQ, Mobility Inventory for Agoraphobia, PSWQ, CSQ-8	Reduction of therapy-related expenses and burdens for patients and their relatives
**9**	**Vivira**	Muscles, bones and joints / koxarthrosis	In publication process	Ib (RCT)	N = 213	Mobile-app “Vivira”; therapeutic home exercise programme	Physiotherapeutic treatment, home exercise instruction	3	Primary: reduction of back pain measured by VNRS; Secondary: quality of life measured by SF-36	
**10**	**vorvida**	Mental disorder / mental and behavioral disorders caused by alcohol	Zill et al. (2019) + Zill et al. (2016) [41, 42]	Ib (RCT)	N = 608 (IG = 306, CG = 302)	Unguided web-based intervention (vorvida)	Waitlist	6	Primary: alcohol consumption measured by QFI, TFB; Secondary: drinking behaviour (binge drinking, drunkenness) measured by Binge drinking, Drunkenness	Patient sovereignty

*CG* control group, *IG* intervention group, *ITT* intention to treat, *LoE* The level of evidence, *RCT* randomised controlled trial, *NA* not available, 53-item *BSI* Brief Symptom Inventory, *AADQ* Acceptance and Action Diabetes Questionnaire, *ACQ* Agoraphobic Cognitions Questionnaire, *APSQ* Anxiety and Preoccupation about Sleep Questionnaire, *BAI* Beck Anxiety Inventory, *BDI* Beck Depression Inventory, *BDI-II* Beck Depression Inventory-II, *BSI* Brief Symptom Inventory, *BSI-Somatization* Somatization subscale of the Brief Symptom Inventory, *BSQ* Body Sensations Questionnaire, *CES-D* Center for Epidemiological Studies’ Depression Scale, *CSQ-8* Client Satisfaction Questionnaire, *DAS* Dysfunctional Attitudes Scale, *DASS-21* Depression Anxiety Stress Scales – Short Form, *DSMQ* Diabetes Self-Management Questionnaire, *ERSQ-27* Emotion Regulation Skills Questionnaire, *FSFI* Female Sexual Functioning Index, *FSMC* Fatigue Scale for Motor and Cognitive Functions, *FSQ* Fear of Sexuality Questionnaire, *GAD-7* Generalized Anxiety Disorder – 7, *HADS-A, HADS-D* Hospital Anxiety and Depression Scales, *HAQUAMS* Hamburg Quality of Life Questionnaire for MS, *HDRS-24* Hamilton Depression Rating Scale, *IIP* Inventory of Interpersonal Problems, *ISI* Insomina Severity Index, *mini-TQ-12*  Mini-Tinnitus Questionnaire, *MSNQ* Multiple Sclerosis Neuropsychological Screening Questionnaire, *PAID* Problem Areas in Diabetes scale, *PEQ* Primary Endpoint Questionnaire, *PHQ-15* Patient Health Questionnaire - 15 items, *PHQ-9* Patient Health Questionnaire, *PSS-10* Perceived Stress Scale-10, *PSQ20* Perceived-Stress-Questionnaire 20 items, *PSWQ* Penn State Worry Questionnaire, *PSWQ-PW* Penn State Worry Questionnaire, Ultra Brief Version-past week, *QFI* The Quantity-Frequency-Index, *QIDS-C16* Quick Inventory of Depressive Symptomatology, *REQ-PD* Recovery Experience Questionnaire, *RSE* Rosenberg Self-Esteem Scale, *SBQ-R* Suicide Behaviors Questionnaire-Revised, *SF-12/36* Short-Form Health Survey- 12 or 36 items, *SIAS* Social Interaction Anxiety Scale, *SPS* Social Phobia Scale, *SRBQ* Sleep-related Behaviours Questionnaire, *SWOP-K9* Self-Efficacy Optimism and Pessimism Questionnaire – 9 items, *TFB* Timeline-Follow-Back, *TiC-P-G* Trimbos and Institute of Medical Technology Assessment Cost Questionnaire for Psychiatry, *UWES* Utrecht Work Engagement Scale, *VNRS* Verbal Numerical Rating Scale, *VPCQ* Vaginal Penetration Cognition Questionnaire, *WHOQOL-BREF* Quality of Life scale

^a^The contents of the table are taken from the DiGA directory [22] and the corresponding publications of trials

^b^ Based on classification according to chapter 2, section 3, § 11, Nr. 2 of the rules of procedure of the Federal Joint Committee [23]

^c^Guided = with professional support

^d^Unguided = without professional support

^e^The information on primary and secondary outcomes are taken from the DiGA directory and from the registry entry with the number DRKS00022973 available at the International Clinical Trials Registry Platform (ICTRP)

Medical outcomes were investigated for all DiHA. In all published and unpublished trials (*n* = 13/13), at least one secondary medical outcome was investigated in addition to the primary medical outcome. Medical outcomes mostly include morbidity such as reduction of the disease symptoms, and improvement of quality of life. Patient-centred structural and procedural effects were investigated in clinical trials of two DiHA (Velibra and Vorvida), including reduced therapy-related costs and burdens for patients and their relatives, and increased patient sovereignty. In three trials (n = 3/13) [26, 36, 40], at least one outcome was measured by different survey instruments. In two trials [29, 38], a survey instrument was used to investigate primary and secondary outcomes. The survey instruments were partly validated instruments, such as Patient Health Questionnaire-9 (deprexis, Kalmeda), the General Depression Scale (HelloBetter Diabetes and Depression), and the Hospital Anxiety and Depression Scale (Elevida).

The intervention of the experimental arm in the clinical trials was either guided, i.e. with professional support via e-mail or other contact, or unguided, i.e. without professional support via e-mail or other contact, web- or mobile-based intervention via application, or care as usual and the application. The control group was either “Waitlist” or “Care as usual”.

## Risk of bias assessment of identified RCTs

**Fig. 5:**
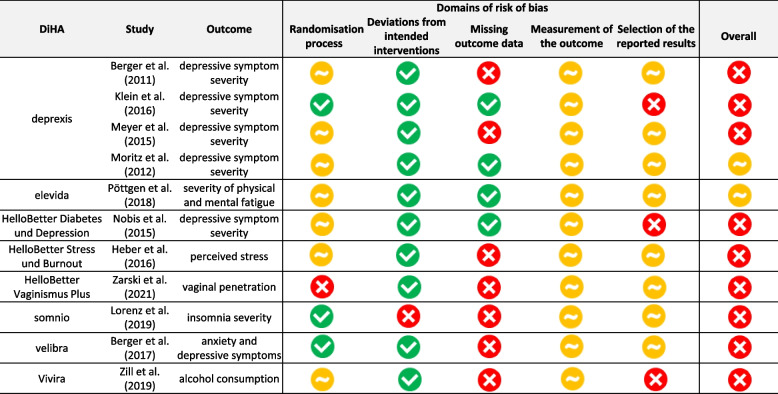
Evaluating risk of bias of included clinical trials (own illustration)

Risk of bias in eleven published RCTs of eight of ten permanently included DiHA was assessed based on the RoB II tool [24]. The agreement rate between the two assessors (HL, HE) across all clinical trials was 93.4%. Clinical trials of two DiHA were at the time of assessment in publication process (Kalmeda and Vivira). Nine of eleven RCTs were judged to be at high risk of bias and two clinical trials raise some concerns. Visual presentations of overall risk of bias per assessment category for included clinical trials can be found in Fig. 5, and a justification of the evaluation in the Additional file 2.

### Randomisation

The randomisation process was associated with a low risk of bias in three clinical trials. These clinical trials showed random allocation sequence generation and concealed allocation. About two-thirds (*n* = 7/11) of the clinical trials were associated with some concerns as no information on the allocation sequence was available. One study [38] resulted in a high risk of bias, as baseline differences between intervention groups suggest a problem with the randomisation process.

### Deviation from the intended intervention

For deviations from intended interventions, almost all clinical trials (*n* = 10/11) had low risk of bias, as no deviation from the intended intervention was observed. Almost all authors conducted an intention-to-treat analysis (*n* = 10/11). The authors of one study [39] did not provide information on the analyses conducted to estimate the effect of assignment to intervention.

### Missing outcome data

For around two-thirds of the clinical trials (*n* = 7/11), there was substantial amount of missing outcome data. There is reason to suggest that missing outcome data is related to its true value, which is why the potential for bias in this category was estimated to be high. Sensitivity analyses was performed for two clinical trials [33, 35]. Those were assessed with a low risk of bias in the domain. In two clinical trials [26, 29], it was not likely that missing outcome data depended on its true value—this led to some concerns.

### Measurement of the outcome

All eleven clinical trials raised some concerns as participants’ knowledge of their assignment to the intervention or control could theoretically lead them to over- or understate their outcome measurements; however, there is no evidence that such bias occurred.

### Selection of the reported results

Almost all of the clinical trials (*n* = 10/11) had a low risk of bias in the selection of the reported results as a pre‐specified protocol was provided and data produced was analysed in accordance with the pre‐specified analysis plan (Additional file 2). Almost one third of the clinical trials (*n* = 3/11) raised some concerns as it remains unclear if selective reporting occurred (Additional file 2). One third of the clinical trials (*n* = 3/11) had a high risk of bias as outcomes that were supposed to be investigated according to the protocol were not mentioned in the study or were not evaluated (Fig. 5).

## Discussion

European countries are struggling to advance DiHA adoption due to several reasons. Germany is the first country in Europe where DiHA have been systematically included into the benefit basket of SHI and therefore can be prescribed by office-based physicians and psychotherapists. With the fast-track pathway aiming at evidence-based inclusion of DiHA into SHI’s benefit basket, DiHA are available to respective patient groups Germany-wide instead of being available only for patients through certain selective programmes of individual sickness funds. Although, the fast-track-pathway can serve as an example for other countries on how to advance the adoption of DiHA with a simultaneous focus on improving health care effects [7], we identified several shortcomings that should be taken into account by policy makers and industry to finally pave the way for evidenced-based decision-making regarding DiHA. (1) Reporting quality in studies often is insufficient. (2) Shortcomings have been identified, e.g. regarding an adequate control group. (3) Patient-centred structural and procedural effects are given only little consideration, although those might be important in real world settings. (4) There is a lack of transparency as to whether and to what extent prices reflect benefits. These points are discussed in more detail below.

About one third of all DiHA listed in the SHI’s directory is permanently listed. A comparative study must have been conducted to prove positive health care effects [43]. Although the manufacturer is free in selecting the study design, the study design depends on the type of DiHA and the health care effects to be evaluated [43]. Our study shows that all permanently included DiHA (*n* = 10) in the directory are based on RCTs (*n* = 13); two are still in the publication process. However, a high risk of bias was found for eleven clinical trials (seven DiHA) and there were some concerns for two clinical trials. In particular, the clinical trials did not score well in terms of missing outcome data and measurement of the outcomes. Regarding the control group, the Federal Association of Sickness Funds criticises that often there is no active control group, but instead a waiting list [44]. Therefore, it is not possible to determine a treatment advantage of the DiHA compared to other DiHA or conventional treatment. Manufacturers argue that a control group that does not receive guideline-compliant therapy would reflect the common care reality in Germany [45]. It is questionable with regard to the comparison group whether an DiHA plus standard treatment or standard treatment alone would be sufficient [46]. A comparison group could be a waiting group approach, a guideline-conform face-to-face treatment or a regular treatment approach, which represents the most promising approach as the other two approaches are ethically less justifiable or represent a clear difference to the DiHA [47]. It would be conceivable to evaluate continuously changing variants of the same DiHA that are constantly compared with each other in a randomised way [48]. However, also the results of the experimental arm could be biased because the authors did not provide information on concomitant treatment. Trial authors must therefore indicate all concomitant treatments that the patients receive during the trial. In addition, the small number of study participants randomised in some clinical trials should be viewed critically, as this can limit the validity of the study [49].

It is remarkable that all clinical trials of the permanently included DiHA reported outcomes with regard to a medical outcome, but only a few evaluate a patient-centred structural and procedural outcomes. This could be due to the fact that the clinical trials were conducted or started before the DiHA legislation, and the operationalisation of patient-centred structural and procedural outcomes is complex [50, 51]. For provisionally listed DiHA, more manufacturers plan to prove patient-centred structural and procedural outcomes [22]. Therefore, increased evaluation of these outcomes can be expected in future, necessitating new study designs and paving the way for the use of real-world data. Using real-world-data and thus alternative study designs in the evaluation of health care effects of DiHA would also have been possible and is also particularly suitable for the evaluation of the frequency of use and compliance of DiHA use. Therefore, guidance documents on the use of real-world data and respective study designs may help to set up appropriate studies. However, an early exchange with the BfArM is recommended in order to plan the evaluation concept accordingly [46]. Until now, new study designs are not yet widely used, but will be relevant in future [52]. There is a need for appropriate study characteristics to support the generation of evidence [19]. Advantages in pragmatic randomized trials can be seen because this study design is feasible and suitable for the characteristics of DiHA [48, 53].

The reimbursement prices for DiHA are characterised by a wide range. This led to disagreements between the SHI and the DiHA-manufacturers, with the Federal Association of Sickness Funds criticising the current legal basis [44]. According to this, the current law place little emphasis on the assessment of the benefit of a DiHA for patients and leads to excessive prices. However, with the new Framework Agreement on negotiation of maximum reimbursement amounts [54], DiHA will in future be grouped according to the indication and their positive health care effect. After calculation of the group specific maximum reimbursement price, the product-specific maximum reimbursement amount applicable from October 2022 will depend both on the DiHA status (conditionally versus permanently included DiHA) and on the number of redeemed prescription codes/activation codes [54]. In addition, the Framework Agreement specifies the calculation of cumulative thresholds, below which reimbursement by SHI becomes possible without an additional negotiation. If the price set by the manufacturer is above the maximum reimbursement price, out-of-pocket payments may arise for the patient.

There are similarities with the procedure for new pharmaceuticals entering the market, e.g. free price setting in the first year, or price negotiations between the manufacturer and the Federal Association of Statutory Health Insurance Funds with valid prices from the 13th month after market approval for pharmaceuticals (DiHA: price negotiations with valid prices from the 13th month after inclusion in the DiHA registry) [55]. However, the transparency of the fast-track assessments of the benefit of DiHA at the BfArM should be improved. Until now it is unclear whether the body of evidence included in the assessment is complete and to what extent the quality of the evidence and the extent of the positive health care effect are included in the reimbursement decision, while pharmaceuticals are subject to price negotiations according to the level of the additional benefit.

The main limitation of this study needs to be mentioned. The quality assessment is based on those clinical trials used by BfArM for decisions on inclusion in the DiHA directory. It is possible that these are not all clinical trials that exist on the corresponding DiHA.

## Conclusion

This is the first study to provide both a descriptive overview of first results of the fast-track pathway to include DiHA into SHI’s benefit basket, to examine the body of evidence on health care effects used in the fast-track evaluation and to assess the quality of these clinical trials. Results show that primarily the medical outcome was evaluated to show health care effects. As the results of the present study are rather sobering regarding trial quality, improvements are required also with respect to the reporting quality in the clinical trials. Furthermore, the use of patient-centred structural and procedural outcomes in addition to medical outcomes should be strengthened, but may require guidance on the use of real-world data and appropriate study designs. In the future it is also important that prices and benefits of DiHA are in reasonable proportion to each other. Although, the fast-track pathway encourages digital innovations and improves patient access to them, in terms of assessing effectiveness and pricing, systematic structures should be implemented to collect and assess respective data for this purpose.

## Supplementary Information

**Additional file 1.** Overview DiHA in the DiHA directory (Status February 2022)

**Additional file 2.** Justification for Risk of Bias.

## Data Availability

All data generated or analysed during this study are included in this published article [and its supplementary information files].
