# Correction: Digital health applications and the fast-track pathway to public health coverage in Germany: challenges and opportunities based on first results

**DOI:** 10.1186/s12913-023-09679-y

**Published:** 2023-06-14

**Authors:** Hendrikje Lantzsch, Helene Eckhardt, Alessandro Campione, Reinhard Busse, Cornelia Henschke

**Affiliations:** 1 Department of Health Care Management, Technische Universität Berlin, Straße des 17. Juni 135, 10623 Berlin, Germany; 2 Berlin Centre of Health Economics Research, Technische Universität Berlin, Berlin, Germany

**Correction**: *BMC Health Services Research* (2022) 22:1182

10.1186/s12913-022-08500-6

Following publication of the original article [1], the authors identified errors in the Abstract, the Keywords and the ‘Selection of the reported results’ subsection.

Corrections (marked in **bold**):

1. Abstract: Since 2020 Digital Health Applications (DiHA, **German DiGA**) in Germany have been undergoing a systematic pathway to be reimbursed by statutory health insurance (SHI) which is attracting attention in other European countries.
2. Keywords: Digital health applications, Digital health technology, **DiHA**, **DiGA**, **Ehealth**, Evidence evaluation
3. ‘Selection of the reported results’ subsection: **Almost all** of the clinical trials (n = **10**/11) had a low risk of bias in the selection of the reported results as a pre-specified protocol was provided and data produced was analysed in accordance with the pre‐specified analysis plan (**Additional file 2**). Almost one third of the clinical trials (n = 3/11) raised some concerns as it remains unclear if selective reporting occurred (**Additional file 2**). One third of the clinical trials (n = 3/11) had a high risk of bias as outcomes that were supposed to be investigated according to the protocol were not mentioned in the study or were not evaluated **(Fig. 5)**.

The original article [1] has been corrected.
