# Supplementary material for: Digital health applications and the fast-track pathway to public health coverage in Germany: challenges and opportunities based on first results
Source: BMC Health Serv Res. 2022 Sep 21;22:1182. doi: 10.1186/s12913-022-08500-6 (PMC9490912; doi:10.1186/s12913-022-08500-6)
Supplement: Supplementary file 1 — Additional file 1. Overview DiHA in the DiHA directory (Status February 2022) [file 12913_2022_8500_MOESM1_ESM.docx]

Additional file 1 Overview DiHA in the DiHA directory (Status February 2022)

| **No.** | **Name** | **DiHA registry inclusion** | **Company** | **Platforms** | **Price details** (Start package + 90 days) |
| --- | --- | --- | --- | --- | --- |
|  |  | | | | |
| 1 | deprexis | 20.02.2021 | GAIA AG, Germany | Web | 297.50 EUR |
| 2 | elevida | 15.12.2020 | GAIA AG, Germany | Web | 743.75 EUR |
| 3 | HelloBetter Diabetes und Depression | 11.12.2021 | GET.ON Institut für Online Gesundheits-trainings GmbH | Web | 599.00 EUR |
| 4 | HelloBetter Stress und Burnout | 18.10.2021 | GET.ON Institut für Online Gesundheits-trainings GmbH | Web | 599.00 EUR |
| 5 | HelloBetter Vaginismus Plus | 04.02.2022 | GET.ON Institut für Online Gesundheitstrainings GmbH | Web | 599.00 EUR |
| 6 | Kalmeda | 25.09.2020 | mynoise GmbH, Germany | Apple App, Google Play | 203.97 EUR |
| 7 | somnio | 22.10.2020 | mementor DE GmbH, Germany | Web | 464.00 EUR |
| 8 | velibra | 01.10.2020 | GAIA AG, Germany | Web | 476.00 EUR |
| 9 | Vivira | 22.10.2020 | Vivira Health Lab GmbH, Germany | Apple App, Google Play | 239.96 EUR |
| 10 | vorvida | 06.05.2021 | GAIA AG, Germany | Web | 476.00 EUR |
|  |  | | | | |
| 11 | CANKADO PRO-React Onco | 03.05.2021 | CANKADO Service GmbH, Germany | Apple App, Google Play, Web | 499.80 EUR |
| 12 | Cara Care für Reizdarm | 26.12.2021 | HiDoc Technologies GmbH | Apple App, Google Play | 718.20 EUR |
| 13 | companion patella | 04.10.2021 | PrehApp GmbH | Web | 345.10 EUR |
| 14 | ESYSTA - Digitales Diabetesmanagement | 04.07.2021 | Emperra GmbH E-Health Technologies, Germany | Apple App, Google Play,  Web | 249.86 EUR |
| 15 | HelloBetter ratiopharm chronischer Schmerz | 18.12.2021 | GET.ON Institut für Online Gesundheitstrainings GmbH | Web | 599.00 EUR |
| 16 | Invirto- Die Therapie gegen Angst | 03.12.2020 | Sympatient GmbH, Germany | Apple App, Google Play | 428.40 EUR |
| 17 | Kranus Edera | 18.12.2021 | Kranus Health GmbH | Apple App, Google Play | 552.00 EUR |
| 18 | M-sense Migräne* | 16.12.2020 | Newsenselab GmbH, Germany | Apple App, Google Play | 219.98 EUR |
| 19 | Mawendo | 09.08.2021 | Mawendo GmbH, Germany | Web | 119.00 EUR |
| 20 | Mika* | 25.03.2021 | Fosanis GmbH, Germany | Apple App, Google Play | 419.00 EUR |
| 21 | Mindable: Panikstörung und Agoraphobie | 29.04.2021 | Mindable Health GmbH, Germany | Apple App, Google Play | 576.00 EUR |
| 22 | neolexon Aphasie | 06.02.2022 | Limedix GmbH | Apple App, Google Play, Web | 487.90 EUR, 487.90 EUR  (further use for additional 90 days) |
| 23 | NichtraucherHelden-App | 03.07.2021 | NichtraucherHelden GmbH, Germany | Apple App, Google Play | 239.00 EUR, 99.00 EUR  (further use for additional 90 days) |
| 24 | Novego: Depressionen bewältigen | 10.10.2021 | IVPNetworks GmbH | Web | 249.00 EUR |
| 25 | Oviva Direkt für Adipositas | 03.10.2021 | Oviva AG | Apple App, Google Play | 345.00 EUR (Start package), 345.00 EUR (further use for additional 90 days) |
| 26 | Rehappy | 29.12.2020 | Rehappy GmbH, Deutschland | Apple App, Google Play | 449.00 EUR (Start package), 299.00 EUR (further use for additional 90 days) |
| 27 | Selfapys Online-Kurs bei Depression | 16.12.2020 | Selfapy GmbH, Germany | Web | 540.00 EUR |
| 28 | Selfapys Online-Kurs bei Generalisierter Angststörung | 19.06.2021 | Selfapy GmbH, Germany | Web | 540.00 EUR |
| 29 | Selfapys Online-Kurs bei Panikstörung | 19.06.2021 | Selfapy GmbH, Germany | Web | 540.00 EUR |
| 30 | zanadio | 22.10.2020 | aidhere GmbH, Germany | Apple App, Google Play | 499.80 EUR |

* Note: After analysis, this DiHA was removed from the DiHA list
