# Supplementary material for: Digital health applications and the fast-track pathway to public health coverage in Germany: challenges and opportunities based on first results
Source: BMC Health Serv Res. 2022 Sep 21;22:1182. doi: 10.1186/s12913-022-08500-6 (PMC9490912; doi:10.1186/s12913-022-08500-6)
Supplement: Supplementary file 2 — Additional file 2. Justification for Risk of Bias. [file 12913_2022_8500_MOESM2_ESM.docx]

Additional file 2 Justification for Risk of Bias

| **First author (publication year)** | Berger (2011) | Berger (2017) | Heber (2016) | Klein (2016) | Lorenz (2019) | Meyer (2015) | Moritz (2012) | Nobis (2013, 2015) | Pöttgen (2018) | Zarski (2018, 2021) | Zill (2016, 2019) |
| --- | --- | --- | --- | --- | --- | --- | --- | --- | --- | --- | --- |
| **DiHA** | deprexis | velibra | HelloBetter | deprexis | somnio | deprexis | deprexis | HelloBetter | elevida | HelloBetter | vorvida |
| **Outcome (time of measurement) that is being assessed for risk of bias** | Primary Outcome | Primary Outcome | Primary Outcome | Primary Outcome | Primary Outcome | Primary Outcome | Primary Outcome | Primary Outcome | Primary Outcome | Primary Outcome | Primary Outcome |
| **1.1 Was the allocation sequence random?** | Y/PY | Y/PY | Y/PY | Y/PY | Y/PY | Y/PY | NI | Y/PY | Y/PY | Y/PY | Y/PY |
| **Comments** |  |  |  |  |  |  | not reported |  |  |  |  |
| **1.2 Was the allocation sequence concealed until participants were enrolled and assigned to interventions?** | NI | Y/PY | NI | Y/PY | Y/PY | NI | NI | NI | NI | NI | NI |
| **Comments** | not reported |  | not reported |  |  | not reported | not reported | not reported | not reported | not reported | not reported |
| **1.3 Did baseline differences between intervention groups suggest a problem with the randomization process?** | PN/N | PN/N | PN/N | PN/N | PN/N | PN/N | PN/N | PN/N | PN/N | Y/PY | PN/N |
| **Comments** |  |  |  |  |  |  |  |  |  |  | Intervention group: pain was more frequently cited as a cause |
| **Risk of bias arising from the randomization process** | Some concerns | Low | Some concerns | Low | Low | Some concerns | Some concerns | Some concerns | Some concerns | High | Some concerns |
| **2.1. Were participants aware of their assigned intervention during the trial?** | Y/PY | Y/PY | Y/PY | Y/PY | Y/PY | Y/PY | Y/PY | Y/PY | Y/PY | Y/PY | Y/PY |
| **Comments** | Concealment of the intervention is not possible; on the other hand, participants may not have been informed about the individual intervention arms. | | | | | | | | | | |
| **2.2. Were carers and people delivering the interventions aware of participants' assigned intervention during the trial?** | Y/PY | Y/PY | Y/PY | Y/PY | PN/N | PN/N | PN/N | Y/PY | PN/N | Y/PY | NI |
| **Comments** | Concealment of the intervention is not possible | Concealment of the intervention is not possible | Concealment of the intervention is not possible | Concealment of the intervention is not possible | automated web based program | automated web based program |  | Concealment of the intervention is not possible |  | Concealment of the intervention is not possible | Concealment of the intervention is not possible |
| **2.3. If Y/PY/NI to 2.1 or 2.2: Were there deviations from the intended intervention that arose because of the trial context?** | PN/N | PN/N | PN/N | PN/N | PN/N | PN/N | PN/N | PN/N | PN/N | PN/N | PN/N |
| **Comments** |  |  |  |  |  |  |  |  |  |  |  |
| **2.4 If Y/PY to 2.3: Were these deviations likely to have affected the outcome?** |  |  |  |  |  |  |  |  |  |  |  |
| **Comments** |  |  |  |  |  |  |  |  |  |  |  |
| **2.5. If Y/PY/NI to 2.4: Were these deviations from intended intervention balanced between groups?** |  |  |  |  |  |  |  |  |  |  |  |
| **Comments** |  |  |  |  |  |  |  |  |  |  |  |
| **2.6 Was an appropriate analysis used to estimate the effect of assignment to intervention?** | Y/PY | Y/PY | Y/PY | Y/PY | PN/N | Y/PY | Y/PY | Y/PY | Y/PY | Y/PY | Y/PY |
| **Comments** | ITT | ITT | ITT | ITT | No ITT | ITT | ITT | ITT | ITT | ITT | ITT |
| **2.7 If N/PN/NI to 2.6: Was there potential for a substantial impact (on the result) of the failure to analyse participants in the group to which they were randomized?** |  |  |  |  | Y/PY |  |  |  |  |  |  |
| **Comments** |  |  |  |  |  |  |  |  |  |  |  |
| **Risk of bias due to deviations from the intended interventions** | Low | Low | Low | Low | High | Low | Low | Low | Low | Low | Low |
| **3.1 Were data for this outcome available for all, or nearly all, participants randomized?** | PN/N | PN/N | PN/N | PN/N | PN/N | PN/N | PN/N | PN/N | PN/N | PN/N | PN/N |
| **Comments** | Dropout rate > 5 % | Dropout rate > 5 % | Dropout rate > 5 % | Dropout rate > 5 % | Dropout rate > 5 % | Dropout rate > 5 % | Dropout rate > 5 % | Dropout rate > 5 % | Dropout rate > 5 % | Dropout rate > 5 % | Dropout rate > 5 % |
| **3.2 If N/PN/NI to 3.1: Is there evidence that the result was not biased by missing outcome data?** | PN/N | PN/N | PN/N | PN/N | PN/N | PN/N | PN/N | Y/PY | Y/PY | PN/N | PN/N |
| **Comments** | No sensitivity analysis performed | No sensitivity analysis performed;  no data on missing participants provided | No sensitivity analysis performed; difference between groups; no data on missing participants provided | No sensitivity analysis performed | No sensitivity analysis performed; difference between groups; no data on missing participants provided | No sensitivity analysis performed | No sensitivity analysis performed | performed sensitivity analyses | performed sensitivity analyses | No sensitivity analysis performed | No sensitivity analysis performed |
| **3.3 If N/PN to 3.2: Could missingness in the outcome depend on its true value?** | Y/PY | Y/PY | Y/PY | Y/PY | Y/PY | Y/PY | Y/PY | NA | NA | Y/PY | Y/PY |
| **Comments** |  |  |  | No patterns recognizable |  |  | No patterns recognizable |  |  |  |  |
| **3.4 If Y/PY/NI to 3.3: Is it likely that missingness in the outcome depended on its true value?** | Y/PY | Y/PY | Y/PY | NA | Y/PY | Y/PY | NA | NA | NA | Y/PY | Y/PY |
| **Comments** | the study authors did not anything to contact the participants |  | Group differences |  | Group differences |  |  |  |  | Group differences |  |
| **Risk of bias due to missing outcome data** | High | High | High | Low | High | High | Low | Low | Low | High | High |
| **4.1 Was the method of measuring the outcome inappropriate?** | PN/N | PN/N | PN/N | PN/N | PN/N | PN/N | PN/N | PN/N | PN/N | PN/N | PN/N |
| **Comments** |  |  |  |  |  |  |  |  |  |  |  |
| **4.2 Could measurement or ascertainment of the outcome have differed between intervention groups?** | PN/N | PN/N | PN/N | PN/N | PN/N | PN/N | PN/N | PN/N | PN/N | PN/N | PN/N |
| **Comments** |  |  |  |  |  |  |  |  |  |  |  |
| **4.3 If N/PN/NI to 4.1 and 4.2: Were outcome assessors aware of the intervention received by study participants?** | Y/PY | Y/PY | Y/PY | Y/PY | Y/PY | Y/PY | Y/PY | Y/PY | Y/PY | Y/PY | Y/PY |
| **Comments** | outcome assessor is the study participant; Study participants knew about their assignment | | | | | | | | | | |
| **4.4 If Y/PY/NI to 4.3: Could assessment of the outcome have been influenced by knowledge of intervention received?** | Y/PY | Y/PY | Y/PY | Y/PY | Y/PY | Y/PY | Y/PY | Y/PY | Y/PY | Y/PY | Y/PY |
| **Comments** |  |  |  |  |  |  |  |  |  |  |  |
| **4.5 If Y/PY/NI to 4.4: Is it likely that assessment of the outcome was influenced by knowledge of intervention received?** | PN/N | PN/N | PN/N | PN/N | PN/N | PN/N | PN/N | PN/N | PN/N | PN/N | PN/N |
| **Comments** |  |  |  |  |  |  |  |  |  |  |  |
| **Risk of bias in measurement of the outcome** | Some concerns | Some concerns | Some concerns | Some concerns | Some concerns | Some concerns | Some concerns | Some concerns | Some concerns | Some concerns | Some concerns |
| **5.1 Were the data that produced this result analysed in accordance with a pre-specified analysis plan that was finalized before unblinded outcome data were available for analysis?** | NI | Y/PY | Y/PY | Y/PY | Y/PY | Y/PY | Y/PY | Y/PY | Y/PY | Y/PY | Y/PY |
| **Comments** | No protocol | Study Register | Study Register (DRKS00004749) | Study Register (NCT01636752+ protocol) | Study Register (NCT02629913) | Study Register (NCT02178631) | Study Register (NCT01401296) | Study Register (DRKS00004748 + protocol) | Study Register (ISRCTN25692173) | Study Register (DRKS00010228 + protocol) | Study Register (DRKS00006104 + protocol) |
| **5.2 Is the numerical result being assessed likely to have been selected, on the basis of the results, from multiple eligible outcome measurements within the outcome domain?** | NI | PN/N | PN/N | Y/PY | PN/N | PN/N | PN/N | Y/PY | PN/N | PN/N | Y/PY |
| **Comments** |  |  |  | 12 months follow-up results not reported |  |  |  | “Behavioural Activation Depression Scale” (BADS) is missing |  |  | “CAEQ4 AASE-G5 RCQ-G6 Readiness-Ruler7 ZUF-88” is missing |
| **5.3 Is the numerical result being assessed likely to have been selected, on the basis of the results, from multiple eligible analyses of the data?** | NI | NI | NI | PN/N | NI | NI | NI | PN/N | NI | Y/PY | NI |
| **Comments** |  |  |  |  |  |  |  |  |  |  | moderators and mediators not assessed |
| **Risk of bias in selection of the reported result** | Some concerns | Some concerns | Some concerns | High | Some concerns | Some concerns | Some concerns | High | Some concerns | Some concerns | High |
| **Overall risk of bias** | High | High | High | High | High | High | Some concerns | High | Some concerns | High | High |
